# Benefits of a Wearable Cyborg HAL (Hybrid Assistive Limb) in Patients with Childhood-Onset Motor Disabilities: A 1-Year Follow-Up Study

**DOI:** 10.3390/pediatric15010017

**Published:** 2023-03-09

**Authors:** Mayumi Matsuda Kuroda, Nobuaki Iwasaki, Hirotaka Mutsuzaki, Kenichi Yoshikawa, Kazushi Takahashi, Tomohiro Nakayama, Junko Nakayama, Ryoko Takeuchi, Yuki Mataki, Haruka Ohguro, Kazuhide Tomita

**Affiliations:** 1 Department of Physical Therapy, Ibaraki Prefectural University of Health Sciences, 4669-2 Ami, Ibaraki 300-0394, Japan; 2 Department of Pediatrics, Ibaraki Prefectural University of Health Sciences Hospital, 4733 Ami, Ibaraki 300-0331, Japan; 3 Center for Medical Science, Ibaraki Prefectural University of Health Sciences, 4669-2 Ami, Ibaraki 300-0394, Japan; 4 Department of Orthopedic Surgery, Ibaraki Prefectural University of Health Sciences Hospital, 4733 Ami, Ibaraki 300-0331, Japan; 5 Department of Physical Therapy, Ibaraki Prefectural University of Health Sciences Hospital, 4733 Ami, Ibaraki 300-0331, Japan; 6 Department of Rehabilitation Medicine, University of Tsukuba Hospital, 2-1-1 Tsukuba, Ibaraki 305-8576, Japan

**Keywords:** exoskeleton device, robotic exoskeleton, exercise therapy, cerebral palsy, walking speed, motor disorders, motor activity, neurological rehabilitation, disabled persons

## Abstract

Rehabilitation robots have shown promise in improving the gait of children with childhood-onset motor disabilities. This study aimed to investigate the long-term benefits of training using a wearable Hybrid Assistive Limb (HAL) in these patients. Training using a HAL was performed for 20 min a day, two to four times a week, over four weeks (12 sessions in total). The Gross Motor Function Measure (GMFM) was the primary outcome measure, and the secondary outcome measures were gait speed, step length, cadence, 6-min walking distance (6MD), Pediatric Evaluation of Disability Inventory, and Canadian Occupational Performance Measure (COPM). Patients underwent assessments before the intervention, immediately after the intervention, and at 1-, 2-, 3-month and 1-year follow-ups. Nine participants (five males, four females; mean age: 18.9 years) with cerebral palsy (*n* = 7), critical illness polyneuropathy (*n* = 1), and encephalitis (*n* = 1) were enrolled. After training using HAL, GMFM, gait speed, cadence, 6MD, and COPM significantly improved (all *p* < 0.05). Improvements in GMFM were maintained one year after the intervention (*p* < 0.001) and in self-selected gait speed and 6MD three months after the intervention (*p* < 0.05). Training using HAL may be safe and feasible for childhood-onset motor disabilities and may maintain long-term improvements in motor function and walking ability.

## 1. Introduction

**Figure 1:**
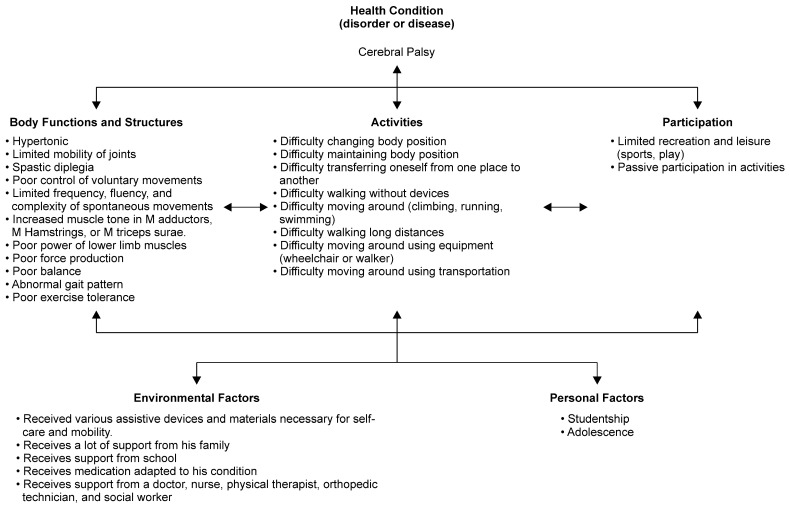
The International Classification of Functioning, Disability and Health-Child and Youth (ICF-CY) framework for cerebral palsy.

Damage to the central nervous system during the developmental process in children leads to various disorders, including motor dysfunction and cognitive impairment, and these impairments progress from childhood to adulthood [1]. The International Classification of Functioning, Disability and Health, Child and Youth (ICF-CY) was suggested by the World Health Organization as a common framework to describe the health and functioning of an individual [2,3]. Therapeutic rehabilitation procedures can enhance body structure and function, and an individual’s activity or participation, according to the ICF-CY. Cerebral palsy (CP), a typical childhood-onset disease, causes physical symptoms such as spasticity, co-contraction of antagonist muscles, muscle weakness, and a lack of selective motor control [4,5]. The ICF-CY model for CP is shown in Figure 1. Of CP patients who were ambulatory in childhood, more than 25% showed a decline in walking ability in early adulthood due to joint pain and gait failure [6,7]. Furthermore, it has been reported that in approximately 30% of adults with CP, the ability to walk is lost before reaching adulthood [8]. Therefore, rehabilitation strategies for the long-term maintenance of motor function are critical for the rehabilitation of children with CP and other motor dysfunctions.

Currently, robot-assisted gait training is increasingly used in the neurorehabilitation of patients with childhood-onset motor disabilities to complement conventional physical therapy [9,10,11,12]. Several robotic devices, such as Gait Trainer™ (Reha-Stim, Berlin, Germany) [9] and Lokomat^®^ (Hocoma, Volketswil, Switzerland) [10,11,12] have been reported to assist gait. In previous studies, robot-assisted gait training improved the ICF domains of ‘body function’ and ‘activity’. Improvements in outcome measures, such as kinematics during gait, gait speed, walking endurance, and gross motor function, were observed in patients with childhood-onset motor disabilities [9,10,11,12].

**Figure 2:**
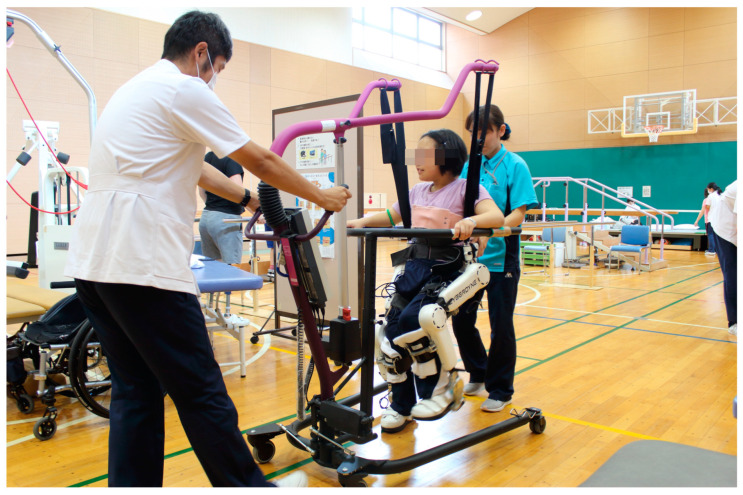
Example of a Hybrid Assistive Limb^®^ (HAL; CYBERDYNE, Tsukuba, Japan) set-up. While walking and wearing the HAL, the patients utilized a walking device (All-in-One Walking Trainer; Healthcare Lifting Specialist, Denmark) with a harness for safety. HAL-assisted training was performed for 2–4 sessions/week for 20 min/session over a 4-week period (12 sessions in total).

Conventional robotic devices enable passive gait training by setting values for gait speed and lower limb joint angles. Thus, even a patient with an entirely paralyzed lower limb can perform gait training by wearing the robotic device. The disadvantage of these passive robotic devices is restricted movement variability for patients. For effective motor learning, kinematic variability during gait and active participation on the part of the patient are crucial [13,14]. Therefore, there is a need for a robotic device that increases kinematic variability and the active participation of the patient during robot-assisted gait training. Thus, we focused our attention on exercise therapy using the lower limb type of the hybrid assistive limb (HAL) (HAL; CYBERDYNE, Tsukuba, Japan) (Figure 2). HAL is a wearable robotic suit that can assist joint motion based on the intention of the wearer [15]. The lower limb type HAL uses information from hip and knee angular sensors, force–pressure sensors in the shoes, and bioelectric signals from electrodes on the extensor and flexor muscles of the knee and hip joints. Based on the information obtained, motion support is determined, and power units located at the hip and knee joints are driven to assist the wearer’s joint motion [16]. Unlike other robots, movement occurs in response to the wearer’s voluntary drive. The effects of training with HAL as an intervention strategy to improve walking performance have been reported in patients with gait disorders of different etiologies for several years [17,18,19,20,21]. In adult patients with stroke and spinal cord injury, improvements in gait speed, step length, cadence, walking endurance, and functional parameters were shown [17,18,19,20,21]; however, HAL is not labeled for use for CP, and this remains investigational. Even when a patient with CP can walk, if the patient continues to walk with an abnormal gait peculiar to CP, such as an equinus or crouching gait, the joints and soft tissues become very stressed. As a result, pain and decreased walking performance sometimes occur during early adulthood [7,8]. For CP, with such abnormal gait patterns, gait training using a HAL is expected to normalize gait and improve walking performance. Gait patterns were compared in patients wearing and not wearing a HAL in CP [22]. When walking while wearing a HAL, compared to walking without a HAL, there was improved single-leg support per gait cycle as well as hip and knee joint angle during gait. Moreover, it was confirmed that gait pattern changes were maintained while walking after removing the HAL. In addition, a HAL is not only a gait-assist robot. It can also be used to practice going up and down stairs, standing, and sitting. Therefore, a HAL can support and improve a wide range of activities, such as standing, sitting, and stair climbing, which are difficult to perform in CP with Gross Motor Function Classification System (GMFCS) levels II to IV.

Patients with CP often have difficulty with the components ‘body function’ and ‘activity’ in the ICF-CY model (Figure 1). However, the HAL uses a new movement support method that assists the wearer’s voluntary motion intentions. It is a wearable movement support robot that is expected to be highly effective not only in improving body functions such as voluntary movement and expanding the range of motion but also in activities such as walking and standing balance performance.

A previous study reported the safety and immediate effects of a single gait training session using a small-sized HAL in adolescent CP patients [22,23]. A further study reported that multiple repetitions of robot-assisted gait training using HAL improved walking ability, walking endurance, and gross motor function in adolescent CP patients [24]. However, with respect to longer-term outcomes, only one case study has reported that improvements in spatiotemporal parameters and lower limb angle in gait were maintained for seven months following the intervention [25]. The long-term effects of robot-assisted gait training using HAL are unclear, not only in young patients with a childhood-onset motor disability but also in adult patients [26,27]. It is also important to clarify its safety and feasibility through long-term follow-up. Additionally, in patients with childhood-onset motor disabilities, it is necessary to clarify the optimal intervention interval and timing based on growth and development.

This study aimed to investigate the long-term benefits of training using a HAL in patients with childhood-onset motor disabilities. Patients underwent long-term follow-ups with assessments before the intervention, immediately after the intervention, and at 1-, 2-, 3-month, and 1-year follow-ups. We were expecting that training using HAL would be useful for a wide range of disorders in childhood-onset.

## 2. Materials and Methods

### 2.1. Patients

This was a single-arm investigation of the intra-individual changes in the patients during the training period and a prospective pre-post study with repeated measurements in the same participants. The study was conducted at our hospital between January 2017 and July 2019. All participants had childhood-onset motor disabilities, were aged ≥10 years, were in GMFCS levels I-IV, understood the study methods, and could fit the lower-limb version of HAL. Height was assumed to be 150–190 cm; however, the limitation to the use of HAL is not height but fit (body-size parameters such as thigh length, lower leg length, and waist width) [10,11,28]. Children with difficulty wearing the HAL due to severe joint deformation and/or contracture; difficulty performing voluntary movements according to instructions because of cognitive dysfunction; in whom the bio-electrodes of the HAL system could not be attached because of skin disease; were judged to be medically unstable by the doctors after comprehensive consideration of physical, blood test, and other findings; or on treatment with botulinum toxin during the previous three months, were excluded [10,11,28].

The study was conducted in accordance with the ethical principles for medical research involving human participants outlined in the Declaration of Helsinki. The study protocol was approved by the ethics committee of Ibaraki Prefectural University of Health Sciences (approval numbers: 682 e83 and e119; approval date: 14 December 2015). All patients and their parents provided written informed consent for their participation in the study and permission for the publication of photographs that might identify them.

### 2.2. The Lower Limb Type HAL

We utilized the lower limb type HAL, size S, which consisted of a lumbar part with batteries, four actuators of the hip and knee, and an integrated exoskeletal frame from the feet to the waist. It was a powered lower-limb wearable robot with a bio-electrical signal control scheme. The component of the intervention using the hybrid control mode used cybernic voluntary control (CVC). However, if bio-electric signals were not detectable because of severe motor disorders, the cybernic autonomous control mode was selected. Sankai et al. reported on these two control systems in detail [15,16].

### 2.3. Study Design

This single-center observational study compared assessment results at six time points. Measurements were performed before and immediately after intervention and at 1-, 2-, 3-month, and 1-year follow-ups after HAL-assisted training. The number of interventions was defined by previous studies on robot training for child patients [11,12,28]. HAL treatment programs were performed for 20 min (excluding intermissions) once a day. In total, the patients underwent 12 training sessions completed over a 4-week period at 2–4 sessions per week. The patients used a mobile suspension system (All-In-One Walking Trainer, Healthcare Lifting Specialist, Denmark) to prevent falling during the walking activity (Figure 2). Gait speed was selected individually by each patient. We performed training using the HAL device in addition to conventional rehabilitation during hospitalization. During hospitalization, physical and occupational therapies were performed five times a week at 40–60 min per session. After discharge, physical or occupational therapy was implemented 0–3 times per month.

### 2.4. Outcome Measures

Outcome measures have been defined previously [24]. All the outcome tools measured difficulty on the activity components of the ICF-CY model for CP (Figure 1). Briefly, we examined the Gross Motor Function Measure (GMFM) [10,11,12,28] as the primary outcome measure. GMFM is an observational evaluation of motor capacity that measures the activity component of the ICF-CY, related to the capacity for movement by changing body position or location, carrying or moving objects, walking, running, or climbing. For secondary outcome measures, walking speed (m/s), stride length (cm), and cadence (steps/min) of self-selected walking speed (SWS) and maximum walking speed (MWS) in the 10 m walking test were measured as indicators of walking performance [9,10,12,17,18,24,29]. The 6-min walking distance (6MD) in the 6-min walking test (6MWT) [9,10,11,24,29] was used as a measure of walking endurance. The 10 m walking test and 6MD were used to measure the “walking” domain of the activity component of the ICF-CY. Additionally, the 6MD was used to measure the “exercise tolerance function” among the body function components of the ICF-CY. The 10 m walking test measured short walking distances, and the 6MD measured long walking distances. The 10 m walking test and 6MD were performed using a walker or bilateral Lofstrand crutches and measured walking performance. The MWS and 6MWT were measured for GMFCS levels I-III. As a performance measure of daily living activities, we used the functional skills scale of the Pediatric Evaluation of Disability Inventory (PEDI) [30]. PEDI measures functional performance in three domains: (1) self-care, (2) mobility, and (3) social function. PEDI measures the “learning and applying knowledge”, “general tasks and demands”, “communication”, “mobility”, “self-care”, “domestic life”, “interpersonal interactions and relationships”, and “community” domains of the activity component of the ICF-CY. We used the Canadian Occupational Performance Measure (COPM) as a measure of the participation component of the ICF-CY [11,31].

### 2.5. Statistical Analysis

Statistical analysis was performed using IBM SPSS Statistics, version 24.0 (IBM Japan, Tokyo, Japan) for each value obtained before the intervention, immediately after the intervention, and at 1-, 2-, 3-month and 1-year follow-ups after wearable robot-assisted training. Differences before and after training were analyzed using the nonparametric Wilcoxon matched-pairs test. Long-term efficacy was tested using one-way repeated measures. The Dunnett test was used as a post-hoc analysis to compare each time result with that of the prior time. The results were compared and examined using average values, with *p* < 0.05 considered statistically significant.

## 3. Results

Nine patients with childhood-onset motor disabilities were enrolled in this study. There were five male and four female patients with a mean age of 18.9 (standard deviation [SD] 6.1; range: 13–32) years, a height of 156.1 (SD 10.8; range: 140–173) cm, and a body weight of 52.6 (SD 11.0; range: 41–78) kg. With respect to GMFCS, there was one level II patient, seven level III patients, and one level IV patient. Diagnoses included CP in seven patients, critical illness polyneuropathy (CIP) in one patient, and encephalitis in one patient. Regarding the paralysis type, six patients presented with spastic diplegia, one with spastic quadriplegia, and two with paraplegia (Table 1).

**Figure 3:**
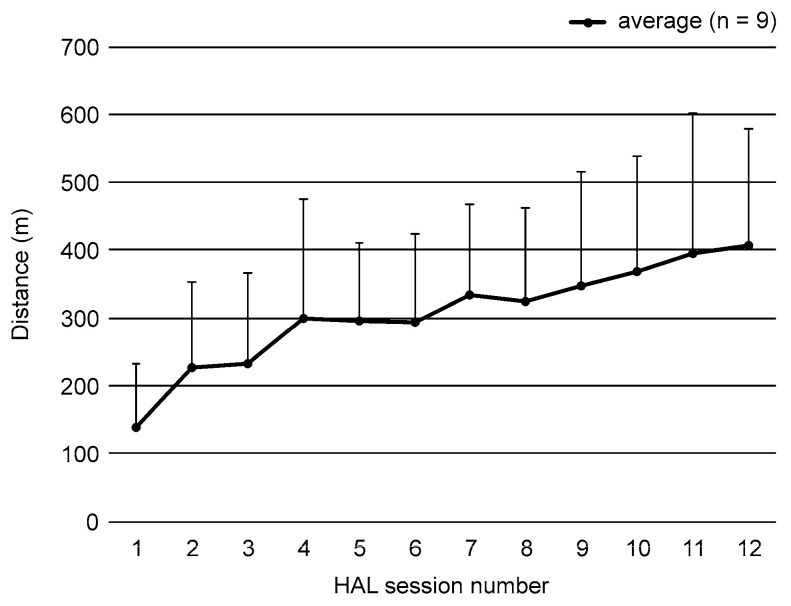
Walking distance results using the Hybrid Assistive Limb^®^ (HAL; CYBERDYNE, Tsukuba, Japan). Each value represents the average walking distance during HAL-assisted training in nine participants. Walking distance significantly increased from the first intervention to the final (12th) HAL-assisted training session.

All nine participants completed the wearable robot-assisted training without adverse events. The average walking distance during the intervention increased significantly from 139 (94) m during the first intervention to 407 (171) m during the final (12th) intervention (Figure 3). For the activity component of the ICF-CY, when the measurements before and after the intervention were compared, significant increases were noted in GMFM (*p* = 0.008), SWS (*p* = 0.015), MWS (*p* = 0.028), cadence during SWS (*p* = 0.015), cadence during MWS (*p* = 0.012), and 6MD (*p* = 0.036) after the training. However, the step length during SWS and MWS did not change after the training (SWS: *p* = 0.214; MWS: *p* = 0.225), and no significant improvement in PEDI (*p* = 1.000) was observed. Regarding the long-term efficacy of the training, there were significant increases in the GMFM immediately after and at the 1-, 2-, 3-month and 1-year follow-ups after the intervention (*p* < 0.001 for all). Additionally, significant improvements were noted in gait speed during SWS immediately, 1, and 3 months after the intervention (*p* = 0.006, 0.015, and 0.013, respectively); in gait speed during MWS immediately and 2 months after the intervention (*p* = 0.009 and 0.025, respectively); in cadence during MWS immediately, 2 months, and 1 year after the intervention (*p* = 0.007, 0.024, and 0.041, respectively); and in 6MD 1 and 3 months after the intervention (*p* = 0.022 and 0.011, respectively) (Table 2).

For the participation component of the ICF-CY, the training resulted in improvements in COPM performance (*p* = 0.024) and satisfaction (*p* = 0.024). Regarding the long-term efficacy of the training, there was an improvement, but it was not significant.

There were some data deficiencies in the measurements. Data loss was observed in COPM 2 months after the intervention in patient 3, 1 month after the intervention in patient 2, and in SWS 1 year after the intervention in patient 9.

## 4. Discussion

The present study indicated that wearable cyborg HAL treatment may be safe and feasible in patients with childhood-onset motor disabilities.

For the activity components of the ICF-CY, gait speed, cadence, and 6MD improvements were maintained 1 and 3 months after the intervention. Furthermore, GMFM was maintained at the 1-year follow-up assessment. The minimal clinically important difference (MCID) by robot-assisted gait training for CP was reported to be 0.1–3.0% (0.26 points—7.92 points) for GMFM [32]. The changes in total GMFM points in these patients were 6.9 points (before–after), 6.7 (before–after 1 month), 7.3 (before–after 2 months), 7.7 (before–after 3 months), and 7.6 points (before–after 1 year), all of which were higher than the minimum MCID. The MCID for 6MD has been reported to be 20–36 m. The changes in 6MD, when compared to the result before the intervention in this study, were 27.1 m (immediately after), 32.4 m (after 1 month), 27.5 m (after 2 months), 35.2 m (after 3 months), and 18.2 m (after 1 year); up to 3 months following the HAL intervention, these values were higher than MCID. The MCIDs of walking performance in the natural history (usual care) over one year in CP have been reported to be +9.1% for gait speed, +5.8% for step length, and +8.1% for cadence [33]. The rates of change one year after HAL intervention were 16.9% for gait speed, 2.1% for step length, and 13.4% for cadence. Gait speed and cadence were significantly higher than MCID. Thus, even the comparison using MCID indicated that the HAL intervention showed improvement in the activity components of the ICF-CY, including GMFM, 6MD, gait speed, and cadence.

It was reported that robot-assisted gait training using HAL improved gait speed, cadence, and walking endurance in adults [17,18,21]. A previous study reported improvements in gait speed, cadence, walking endurance, and GMFM following robot-assisted gait training using HAL [24]. However, these studies reported on the short-term effects of robotic training. There are only a few reports on the long-term effects of training using HAL in patients with childhood-onset motor disabilities and adults [19,21]. One case report which described adult patients with muscular dystrophy reported that gait speed was maintained six months after the intervention. However, walking endurance decreased to pre-intervention values at six months [34]. Conversely, the long-term effects of robot-assisted gait training using conventional devices have been reported in children. In particular, studies on Lokomat have reported that gait speed, 6MD, GMFM (activity component on the ICF-CY), and COPM (participation component on the ICF-CY) were maintained at two and six months in pediatric patients [11,12]. A study on the effects of Gait Trainer on pediatric patients reported that gait speed and 6MD (activity component on the ICF-CY) were maintained one month after the intervention [9]. HAL functions as if it were part of the body according to the wearer’s motor intentions and ideal internal movement patterns [35]. It is important that the generalization of human movement is accompanied by the patient’s voluntary intention. The conventional robot with full and constant guidance often leads to patients being passive [36]. HAL differs from conventional robots. Passive assistance reduces the patient’s effort, thereby weakening the motor learning effect [37]. Furthermore, active participation, dynamic walking pattern adaptation, and variability in movements are essential to improve motor learning [38,39]. HAL was a wearable cyborg that wearer could walk on the ground, not only on a treadmill. Comparing treadmill walking with a robotic device that could walk on the ground with body weight support, young neurological patients reported greater stride-to-stride variability and inter-joint kinematics variability when walking on the ground, as well as requiring more concentration than walking on a treadmill [40]. Therefore, HAL training capable of walking on the ground may have required the patient to navigate the floor and need to plan and adjust gait patterns to cope with the demands of the environment, and the patients’ feedback mechanisms may have worked more. Therefore, wearable robot training using HAL for patients with childhood-onset motor disabilities may have a longer-term effect than training with conventional robotic devices, such as Lokomat and Gait Trainer. Since robotic rehabilitation is a method of motor learning, it is generally considered that Hebb’s law can be applied. However, further investigation is needed to determine at what point the learning effect of HAL training reaches a plateau, depending on the amount and duration of intervention. In this regard, observational studies have begun in rare neuromuscular diseases [41].

Spasticity is the most common sign of abnormal muscle tone in patients with CP. Muscle spasticity, impaired balance, and other motor control deficits reduce walking efficiency and increase energy requirements, even if a CP patient with GMFCS level II can walk outdoors [42]. The decline of GMFCS levels has been reported for patients aged ≤18 years [43]. It has been reported that even ambulatory young adults with CP experience decreased walking ability at an early age [7,8]. Even in ambulatory CP, patients have a crouching posture when standing and walking. Gait speed and walking endurance are reduced because walking efficiency is greatly reduced by the crouching posture. Therefore, patients with GMFCS level II CP use Lofstrand crutches for daily transfer but experience difficulty walking long distances. Because the activity component of the ICF-CY was improved by the HAL intervention, the possibility of extending walking distance using the HAL intervention, even in ambulatory CP, was indicated.

A plateau in motor function in CP is reached at the age of seven years, and in severe CP below GMFCS level III, secondary functional decline occurs after the age of seven [36]. Most of our participants had adolescent CP with GMFCS level III and therefore had a high risk of secondary functional decline in their natural course. Our participants continued with one or two outpatient physical therapy sessions per month for one year after the HAL intervention. All participants were wheelchair users. In detail, participants with GMFCS level II moved using walkers only in the school buildings. Participants with GMFCS levels III and IV walked only during the physical therapy sessions. These results suggest that the beneficial effects of wearable robot-assisted training on the walking performance of patients with childhood-onset motor disabilities who walk very infrequently after the intervention may be maintained for three months. Further, our results suggest that gross motor function may be maintained for a longer period (more than one year).

Regarding the optimal amount and timing of robot training using Locomat in stroke patients have reported that younger age, early intervention after injury, and higher training doses (at least 14 sessions) are associated with better outcomes [44]. In the present study of young neurological patients, fewer doses (12 sessions) could lead to statistically significant differences in walking ability and gross motor function. Considering the brain’s pathophysiology, the number of task-specific repetitions built on motor learning increases may increase the amount of improved functional outcomes. Further, the benefit that could be gained may be expected by increasing the number of interventions in the future.

Another advantage of wearable robot-assisted training using HAL is that improvements have been noted with fewer repetitions in children than in adult patients [18,21]. In this study, as few as 12 repetitions of the training clearly produced improvements in walking ability and gross motor function. However, reports showed that over 50 repetitions of HAL-assisted training were required for improvements in adult patients with spinal cord injury who had bilateral lower limb paralysis and who were similar to the patients in this study with childhood-onset motor disabilities [18,21]. Therefore, the results suggest that adaptation to HAL-assisted training occurs earlier in children than in adults. The intervention using the HAL was an opportunity for child patients to learn normal walking motion, and it appeared to indicate improvement in walking performance and GMFM (the activity component of the ICF-CY). In addition, this case included not only CP but also other neurological patients, such as CIP and encephalitis. Hubertus J.A. Van Hedel et al. [40,45] reported the effects of gait training using a robotic device in young neurological patients with CP as well as CIP, hemiparesis, and demyelination. In addition, robot-assisted gait therapy is increasingly being used in pediatric neurorehabilitation for neurological gait disorders, including meningomyelocele (spina bifida) as well as CP, complementing conventional physical therapy [13]. In this study, improvement in motor function and walking ability after HAL training was observed not only in CP but also in CIP and encephalitis. The target disorders for pediatric rehabilitation are various. This study with younger neurological patients with a heterogeneous population reflected everyday clinical situations. Therefore, robotic rehabilitation may be useful not only for CP but also for childhood-onset movement disorders.

This study has several limitations. There are no control subjects in this study. Moreover, the sample size was small, and our patient group was heterogeneous regarding diagnosis, age, and motor skills. Therefore, the generalizability of the findings may be limited. Among patients with childhood-onset motor disabilities, it is difficult to isolate a uniform group of patients because of large variations in medical conditions and motor functions. Nakajima et al. reported the efficacy of HAL treatment in patients with slowly progressive rare neuromuscular diseases using a controlled crossover trial [35]. In the future, comparative tests with a larger study population and a control group are needed to investigate potential differences in the intervention effect due to age and severity of motor disabilities. In addition, although the long-term effects of HAL intervention were examined, there was no evaluation between the 3-month and 1-year follow-up intervals. Further, only patients who could use the S-size HAL were included. In the future, the effects of wearable robot-assisted training HAL for patients with childhood-onset motor disabilities should be investigated in a wide range of age groups.

## 5. Conclusions

Wearable robot training for patients with childhood-onset motor disabilities may be safe and feasible. Our results suggest that wearable robot training may maintain improvements in walking performance and endurance for 3 months post-intervention and motor function improvement for 1-year post-intervention. Further, wearable robot training using HAL may be an effective intervention for patients with childhood-onset motor disabilities.

## Figures and Tables

**Table 1:** Characteristics of patients and Hybrid Assistive Limb^®^ settings.

Patient	Sex	Age (Years)	Height (cm)	Weight (kg)	Etiology	Paralysis Type	GMFCS	Walking Distance/ Session (m), Median (Range)	HAL Mode of Action (Hip/Knee)
1	M	16	160	45	CP	SD	II	570 (200–840)	CVC/CVC
2	M	32	173	61	CIP	SP	III	192 (40–280)	CVC/CVC
3	M	24	160	49	CP	SD	III	390 (200–520)	CVC/CVC
4	F	22	156	41	CP	SD	III	233 (120–280)	CVC/CVC
5	M	17	153	51	CP	SD	III	350 (240–480)	CVC/CVC
6	F	17	140	49	CP	SD	III	362 (80–520)	CVC/CVC
7	F	15	168	78	Encephalitis	SP	III	183 (40–320)	CAC/CVC
8	F	13	142	48	CP	SD	III	249 (80–420)	CVC/CVC
9	M	14	153	51	CP	SQ	IV	220 (80–320)	CVC/CVC
Average		18.9	156.1	52.6					
Standard deviation		6.1	10.8	11.0					

Abbreviations: M, male; F, female; CP, cerebral palsy; CIP, critical illness polyneuropathy; SD, spastic diplegia; SP, spastic paraplegia; SQ, spastic quadriplegia; GMFCS, Gross Motor Function Classification System; CVC, Cybernic Voluntary Control; CAC, Cybernic Autonomous Control. Hybrid Assistive Limb^®®^ (HAL, CYBERDYNE, Tsukuba, Japan).

**Table 2:** Outcome measures before and after Hybrid Assistive Limb^®®^ (HAL, CYBERDYNE, Tsukuba, Japan) training sessions.

Outcome Measure		*n*	Time								
			Before	After	1 Month after	2 Months after	3 Months after	1 Year after			
			Mean (±SD)	Mean (±SD)	Mean (±SD)	Mean (±SD)	Mean (±SD)	Mean (±SD)	F	*p*	η^2^
Motor function	GMFM total (score)	9	159.0 ± 17.4	165.9 ± 20.3 ^a,d^	165.7 ± 20.7 ^d^	166.3 ± 21.8 ^d^	166.7 ± 20.7 ^d^	166.6 ± 21.7 ^d^	8.713	<0.001	0.52
Walking ability	SWS gait speed (m/s)	9	0.46 ± 0.24	0.62 ± 0.42 ^a,c^	0.61 ± 0.35 ^b^	0.57 ± 0.34	0.61 ± 0.41 ^b^	0.59 ± 0.32	3.671	0.009	0.34
	step length (cm)	9	41.5 ± 10.0	44.8 ± 11.3	45.6 ± 10.0	43.5 ± 9.7	45.2 ± 10.2	44.0 ± 5.8	2.926	0.026	0.30
	cadence (steps/min)	9	65.4 ± 29.0	78 ± 38.9 ^a,b^	77.2 ± 37.9	77.1 ± 36.4	76.5 ± 41.1	78.6 ± 39.3	3.005	0.023	0.30
	MWS gait speed (m/s)	8	0.65 ± 0.34	0.83 ± 0.49 ^a,c^	0.78 ± 0.38	0.81 ± 0.44 ^b^	0.77 ± 0.38	0.76 ± 0.43	2.802	0.031	0.29
	step length (cm)	8	46.9 ± 8.2	49.2 ± 9.2	49.5 ± 8.4	49.1 ± 9.0	49.7 ± 9.5	47.9 ± 8.8	0.950	0.461	0.12
	cadence (steps/min)	8	82.0 ± 38.9	95.7 ± 46.9 ^a,c^	92.5 ± 39.8	93.9 ± 39.6 ^b^	91.3 ± 39.0	93.0 ± 46.0 ^b^	3.016	0.023	0.30
Walking endurance	6MD (m)	8	161.4 ± 67.1	188.5 ± 84.6 ^a^	193.8 ± 87.9 ^b^	188.9 ± 73.5	196.6 ± 84.8 ^b^	179.6 ± 81.8	2.925	0.026	0.30
Subjective evaluation	COPM performance (score)	9	3.4 ± 0.9	4.4 ± 1.3 ^a^	4.5 ± 1.4	3.9 ± 1.4	3.8 ± 1.7	4.2 ± 1.5	0.595	0.704	0.09
	COPM satisfaction (score)	9	2.9 ± 1.2	3.9 ± 1.3 ^a^	4.0 ± 1.5	3.5 ± 1.5	3.8 ± 1.7	3.8 ± 1.6	0.914	0.485	0.13
ADL	PEDI (score)	9	159.6 ± 30.5	159.6 ± 30.5	159.8 ± 30.5	159.9 ± 30.6	160.0 ± 30.6	162.4 ± 30.7	1.759	0.147	0.20

Comparison before and after HAL-assisted training using Wilcoxon matched-pairs signed-ranks test, ^a^ *p* < 0.05. The persistence of the effect was tested using one-way repeated measures ANOVA and post hoc Dunnett tests, ^b^ *p* < 0.05, ^c^ *p* < 0.01, ^d^ *p* < 0.001. Abbreviations: SWS, self-selected walking speed; MWS, maximum walking speed; 6MD, 6-min walking distance; GMFM, Gross Motor Function Measure; COPM, Canadian Occupational Performance Measure; PEDI, Pediatric Evaluation of Disability Inventory.

## Data Availability

Not applicable.
